# Engineered Heusler Ferrimagnets with a Large Perpendicular Magnetic Anisotropy

**DOI:** 10.3390/ma8095320

**Published:** 2015-09-22

**Authors:** Reza Ranjbar, Kazuya Suzuki, Atsushi Sugihara, Terunobu Miyazaki, Yasuo Ando, Shigemi Mizukami

**Affiliations:** 1 WPI-Advanced Institute for Materials Research, Tohoku University, Sendai 980-8577, Japan; E-Mails: kazuya.suzuki.fw@wpi-aimr.tohoku.ac.jp (K.S.); a.sugihara@wpi-aimr.tohoku.ac.jp (A.S.); miyazaki@wpi-aimr.tohoku.ac.jp (T.M.); mizukami@wpi-aimr.tohoku.ac.jp (S.M.); 2 Department of Applied Physics, Graduate School of Engineering, Tohoku University, Sendai 980-8579, Japan; E-Mail: ando@mlab.apph.tohoku.ac.jp

**Keywords:** epitaxial film, MnGa, Co_2_MnSi cubic Heusler alloy, synthetic PMA ferrimagnets

## Abstract

Synthetic perpendicular magnetic anisotropy (PMA) ferrimagnets consisting of 30-nm-thick *D*0${}_{22}$-MnGa and Co${}_{2}$MnSi (CMS) cubic Heusler alloys with different thicknesses of 1, 3, 5, 10 and 20 nm, buffered and capped with a Cr film, are successfully grown epitaxially on MgO substrate. Two series samples with and without post annealing at 400 ${}^{\circ }$C are fabricated. The (002) peak of the cubic *L*2${}_{1}$ structure of CMS films on the MnGa layer is observed, even for the 3-nm-thick CMS film for both un-annealed and annealed samples. The smaller remnant magnetization and larger switching field values of CMS (1–20 nm)/MnGa (30 nm) bilayers compared with 30-nm-thick MnGa indicates antiferromagnetic (AFM) interfacial exchange coupling (*J*${}_{\mathrm{ex}}$) between MnGa and CMS films for both un-annealed and annealed samples. The critical thickness of the CMS film for observing PMA with AFM coupling in the CMS/MnGa bilayer is less than 10 nm, which is relatively large compared to previous studies.

## 1. Introduction

A bilayer structure consisting of magnetic films with perpendicular magnetic anisotropy (PMA) and in-plane magnetic anisotropy exhibit full PMA when interfacial exchange coupling (*J*${}_{\mathrm{ex}}$) is sufficiently strong. Such bilayers with negative *J*${}_{\mathrm{ex}}$, *i.e.*, synthetic PMA ferrimagnets, are practically important for perpendicular magnetic tunnel junctions (p-MTJs) for high density spin-transfer-torque magnetoresistive random access memory (STT-MRAM) [1,2]. In general, PMA magnetic materials should have large magnetic anisotropy, a low damping constant, small saturation magnetization, and high spin polarization. These properties are essential to optimize the thermal stability factor and critical current density in STT-MRAM [3,4]. Tetragonal MnGa alloys have two crystal structures: *L*1${}_{0}$ and *D*0${}_{22}$, which depends on the Mn composition. *L*1${}_{0}$ ordered structures exhibit good stability when the content of Mn is approximately 50%–65%, while *D*0${}_{22}$ structures appear and exhibit strong ferrimagnetism when the Mn content is approximately 65%–75% [5]. To date, many studies have focused on structural, magnetic, and transport properties, spin polarization, and magnetization dynamics of MnGa alloys [5,6,7,8,9,10,11,12,13,14,15,16,17]. Based on these studies, MnGa alloys have a small and tunable saturation magnetization of approximately 200–600 emu/cm${}^{3}$, a large uniaxial magnetic anisotropy (*K*${}_{u}$) value of approximately 10–15 Merg/cm${}^{3}$, a high spin polarization of approximately 0.4–0.58, a high Curie temperature above 730 K, and a low Gilbert damping constant less than 0.01. Although this material shows a very small tunneling magnetoresistance (TMR) ratio in p-MTJs, which is not comparable with the predicted TMR value (600%) reported by Kubota *et al.* [18], this material has potential to be used as a PMA magnetic film in synthetic PMA ferrimagnets. There are few reports concerning the bilayer structure of MnGa as PMA material and ultra-thin three-dimensional ferromagnetic metals/alloys such as Co [19], Fe [20], and Fe-Co [21], which significantly enhance the TMR ratio. Interestingly, Ma *et al.* recently reported that *J*${}_{\mathrm{ex}}$ of the bilayer of *L*1${}_{0}$-MnGa and Co-rich Fe-Co is antiferromagnetic (AFM) with a strength of −3.0 erg/cm${}^{2}$ and tunnel magnetoresistance of 60% (120%) at room (low) temperature [21]. Other candidates in addition to three-dimensional ferromagnetic metals/alloys are Co-based cubic Heusler alloys. Half-metal properties, a large TMR ratio greater than 100%, even at room temperature [22,23], and a low damping constant of Co-based Heusler alloys make these materials interesting in this area. However, there are no reports on such bilayers. We recently reported epitaxy of bilayer films consisting of 30-nm-thick tetragonal Heusler-like *D*0${}_{22}$-MnGa and 20-nm-thick cubic Co-based Heusler alloys including Co${}_{2}$FeAl (CFA), Co${}_{2}$FeSi (CFS), and Co${}_{2}$MnSi (CMS) on (100) single crystalline MgO substrates at different annealing temperatures [24,25]. AFM *J*${}_{\mathrm{ex}}$ was observed in CMS/MnGa and CFS/MnGa bilayers, whereas both ferromagnetic and AFM *J*${}_{\mathrm{ex}}$ were observed in CFA/MnGa bilayers depending on the annealing temperature. A relatively strong *J*${}_{\mathrm{ex}}$ value of approximately −3.2 erg/cm${}^{2}$ was obtained for the CMS/MnGa bilayer annealed at 400 ${}^{\circ }$C [25], which is comparable with that of the Fe-Co/MnGa bilayer [21]. Furthermore, a very good *L*2${}_{1}$ ordered structure of CMS was confirmed for the CMS/MnGa bilayer annealed at 400 ${}^{\circ }$C by a high-resolution transmission electron microscopy (HRTEM) measurement [25]. Therefore, the CMS/MnGa bilayer is adventageous for synthetic PMA ferrimagnets. Herein, we will investigate the effects of the CMS thickness on structural and magnetic properties of the CMS/MnGa bilayer.

## 2. Experimental Design

The CMS (*t*${}_{\mathrm{CMS}}$ = 0, 1, 3, 5, 10 and 20 nm)/*D*0${}_{22}$-MnGa (30 nm) bilayer films were epitaxially grown using an ultrahigh vacuum magnetron sputtering system with a base pressure of <10${}^{-7}$ Pa on a MgO (100) substrate buffered by a 10-nm-thick Cr buffer layer. The Cr, Mn${}_{55}$Ga${}_{45}$ and Co${}_{50}$Mn${}_{25}$Si${}_{25}$ targets with growth rate of 0.852, 0.393 and 0.416 Å/sec were used for deposition of these films, respectively. Pressure of Ar gas was 0.1 Pa. All bilayer films were capped by a 5-nm-thick Cr protection layer. In-situ annealing was performed at 400 ${}^{\circ }$C after MnGa deposition. To investigate the annealing temperature effect, ex-situ annealing was employed at 400 ${}^{\circ }$C using a rapid thermal annealing (RTA) system for the same stacking structure. The actual composition of MnGa and CMS films was examined using an inductively coupled plasma spectroscopy. The film compositions of MnGa and CMS were Mn${}_{70}$Ga${}_{30}$ and Co${}_{45}$Mn${}_{25}$Si${}_{30}$, respectively. To characterize the structural properties, an X-ray diffractometer with Cu Kα radiation was used. Magnetic properties were measured by a vibrating sample magnetometer (VSM) and a polar magneto optical Kerr effect (P-MOKE) system operating at a laser wavelength of 400 nm.

## 3. Results and Discussion

**Figure 1:**
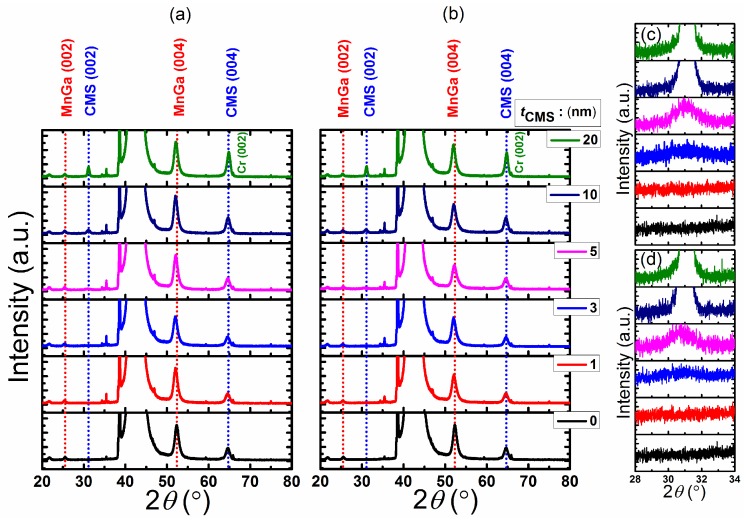
(**a**) X-ray diffraction (XRD) pattern of Co${}_{2}$MnSi (CMS) (0–20 nm)/MnGa (30 nm) bilayers for the un-annealed samples; and (**b**) samples annealed at 400 ${}^{\circ }$C; (**c**) Expanded XRD pattern of CMS (0–20 nm)/MnGa (30 nm) bilayers for the un-annealed samples and (**d**) samples annealed at 400 ${}^{\circ }$C.

**Figure 2:**
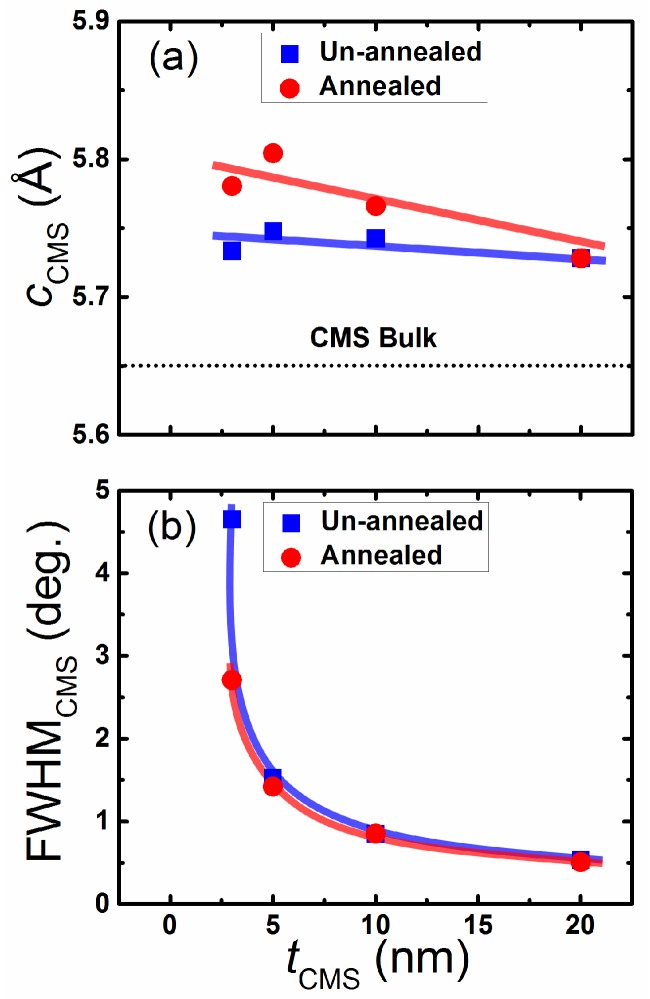
Thickness dependence of (**a**) the *c* lattice constant and (**b**) the full width at half maximum (FWHM) for CMS films.

The out-of-plane X-ray diffraction (XRD) pattern of CMS (0–20 nm)/MnGa (30 nm) bilayers for the un-annealed samples and samples annealed at 400 ${}^{\circ }$C are shown in Figure 1a,b, respectively. The peaks of MnGa (002) and (004) corresponding to the tetragonal structure of *D*0${}_{22}$-MnGa can clearly be observed in both series samples. The *c* lattice constant of 30-nm-thick MnGa film without the CMS film is 7.00 Å. By inserting the CMS film and increasing its thickness between the MnGa film and Cr cap layer, the *c* lattice constant of the MnGa film does not change. The (002) and (004) peaks of the cubic *L*2${}_{1}$ structure of the CMS film can be seen in these figures for the un-annealed and annealed CMS (20 nm)/MnGa (30 nm) bilayer films. However, the (004) peak is superposed on the (002) peak of the Cr layer. In this study, to investigate the structural properties of CMS (0–20 nm)/MnGa (30 nm) bilayers, the (002) peak was considered. As the thickness of CMS decreases, the intensity of the (002) peaks of the cubic structure of the CMS films decreases. The minimum thickness of the CMS film to detect the (002) peak of the cubic structure in an XRD pattern is 3 nm for both un-annealed and annealed samples as shown in Figure 1c,d, respectively. The *t*${}_{\mathrm{CMS}}$ dependence of the *c* lattice constant of CMS films estimated by fitting the (002) peak of the cubic structure of CMS for samples with and without annealing is shown in Figure 1a. This dependence shows a linear reduction with increasing thickness. The *c* lattice of all films is larger than the bulk value, which implies that the cubic structure of the CMS film slightly changes to a tetragonal structure for better matching between the MnGa film and CMS layer. The *c* lattice constant of the bulk value of the CMS alloy is denoted by the dotted line. To estimate the mismatch value between the MnGa film and CMS layer, an in-plane XRD measurement was performed for the un-annealed CMS (20 nm)/MnGa (30 nm) bilayer. The *a* lattice constant of the MnGa and CMS films are 3.937 and 5.567 Å, respectively. An estimated mismatch value of approximately 0.013% is relatively smaller than the bulk value (2.17%), which indicates that the lattice of CMS contracts to fit the MnGa lattice on the bottom in the CMS (20 nm)/MnGa (30 nm) bilayer. The same behavior is expected for the other samples as well. The *t*${}_{\mathrm{CMS}}$ dependence of the full width at half maximum (FWHM) for the (002) peak of the CMS film is shown in Figure 2b for both un-annealed and annealed samples. The dependence shows a nonlinear reduction with increasing thickness for both un-annealed and annealed samples which is consistent with Scherrer’s formula. According to Scherrer’s formula, larger FWHM is expected for thinner films. Furthermore, the smaller FWHM of annealed samples compared with un-annealed samples is also attributed to better crystallization of CMS film after annealing.

**Figure 3:**
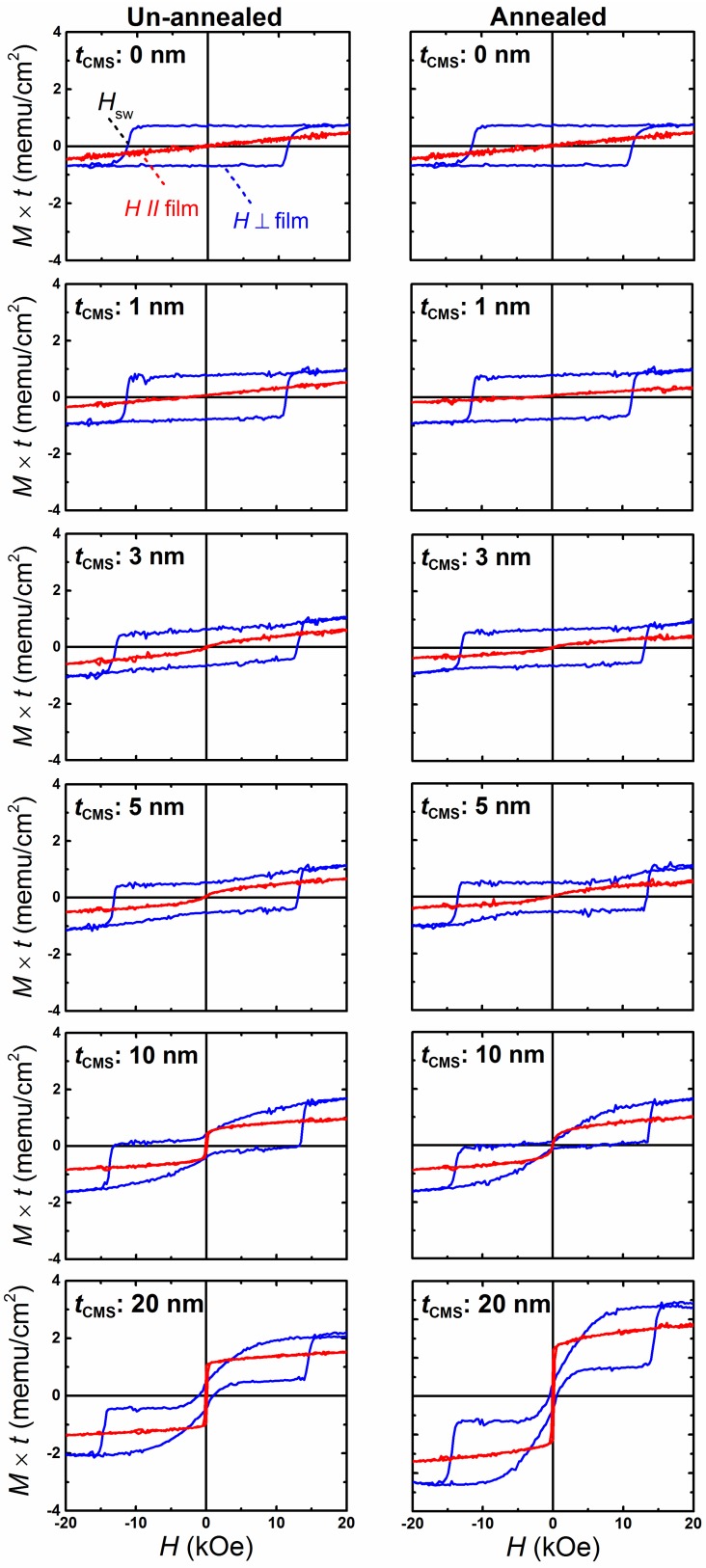
Typical out-of-plane and in-plane magnetization versus applied magnetic field (*M*-*H* loop) of CMS (0–20 nm)/MnGa (30 nm) bilayers for the un-annealed (left figures) and annealed (right figures) samples measured by vibrating sample magnetometer (VSM).

The typical out-of-plane and in-plane magnetization versus applied magnetic field (*M*-*H* loop) of CMS (0–20 nm)/MnGa (30 nm) bilayers measured by VSM for all un-annealed and annealed samples are shown in Figure 3. Both un-annealed and annealed samples with 1-nm-thick CMS exhibit a similar shape to the *M*-*H* loop with the 30-nm-thick MnGa film without the CMS layer as the reference film. By increasing the CMS thickness to 5 nm, no in-plane components are observed at 0 kOe for both series samples. The shape of the *M*-*H* loops of bilayers with 10 and 20 nm thick CMS films changes for both un-annealed and annealed samples in which the in-plane component of magnetization at 0 kOe is clearly observed for these bilayers. The remnant magnetization ($M\times t_{\left(\mathrm{0kOe}\right)}$) and switching magnetic field (*H*${}_{\mathrm{SW}}$) values of both un-annealed and annealed CMS (1–20 nm)/MnGa (30 nm) bilayers are different with a 30-nm-thick MnGa reference film; indeed, the MnGa film and CMS layer are hard and soft magnetic materials, respectively. Hence, the resultant magnetization direction of the CMS/MnGa bilayer film changes because of the interaction in their interfaces. *J*${}_{\mathrm{ex}}$ plays a very important role in this type of bilayer. Suppose that there is no *J*${}_{\mathrm{ex}}$ between the hard and soft layers; in this case, the $M\times t_{\left(\mathrm{0kOe}\right)}$ and *H*${}_{\mathrm{SW}}$ values of the bilayer are the same as those for the hard magnetic film. On the other hand, the $M\times t_{\left(\mathrm{0kOe}\right)}$ and *H*${}_{\mathrm{SW}}$ values of the bilayer are smaller and larger, respectively, than those of the hard magnetic film for the AFM exchange coupling, whereas opposite trends are expected for a ferromagnetic exchange coupling as reported in a previous study [27]. *J*${}_{\mathrm{ex}}$ has been evaluated from a magnetic hysteresis loop for the un-annealed and annealed CMS (20 nm)/MnGa (30 nm) bilayers by using the following equation [25,26]:

(1) $$H_{S\pm }=\pm 4\pi M_{\mathrm{eff}}^{\mathrm{CMS}}-\left(J_{\mathrm{ex}}/M_{S}^{\mathrm{CMS}}t_{\mathrm{CMS}}\right)$$

where $M_{S}^{\mathrm{CMS}}$ and $t_{\mathrm{CMS}}$ are the saturation magnetization and thickness of the CMS film, respectively and 4$\pi M_{\mathrm{eff}}^{C\mathrm{MS}}$ is also effective demagnetization field. $H_{S+}$ and $H_{S-}$ are the saturation fields in the parallel and antiparallel states of the magnetization of the MnGa and CMS films, respectively. Interestingly, a very strong AFM $J_{\mathrm{ex}}$ was obtained for both samples. The evaluated $J_{\mathrm{ex}}$ values were −2.5 and −3.2 erg/cm${}^{2}$ for the un-annealed and annealed CMS (20 nm)/MnGa (30 nm) bilayers, respectively, which are comparable to those of the FeCo/MnGa interface. A large value of $H_{S+}$ and $H_{S-}$ are expected when the AFM $J_{\mathrm{ex}}$ is stronger and also the thickness of CMS is smaller. Bilayers annealed at 400 ${}^{\circ }$C showed enhancement of $H_{S-}$ compared with un-annealed bilayers. Therefore, stronger *J*${}_{\mathrm{ex}}$ value is expected for these samples which is may be attributed to improvement of interfacial quality of CMS film because of annealing as can be seen from XRD result. The $J_{\mathrm{ex}}$ cannot be evaluated for thinner CMS films because the magnetization of the bilayer film is not saturated, even when 20 kOe is applied to the magnetic field. Indeed, a high field measurement is necessary to obtain $H_{S+}$ for evaluation of $J_{\mathrm{ex}}$ of thinner CMS films in which the 4$\pi M_{\mathrm{eff}}^{C\mathrm{MS}}$ is also unknown.

**Figure 4:**
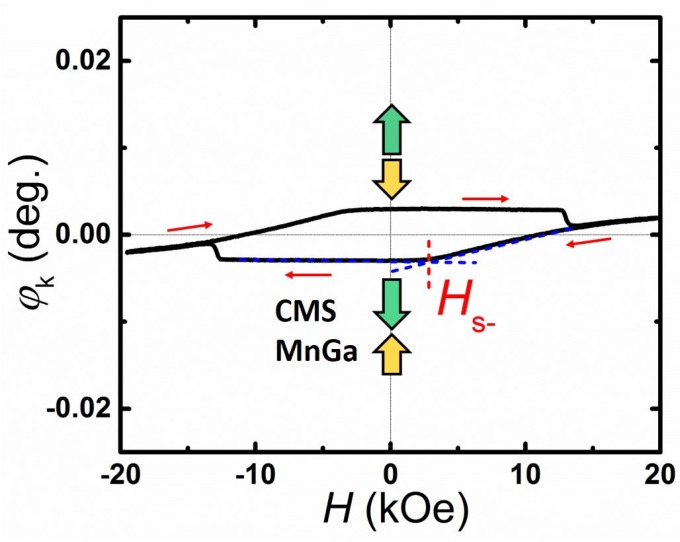
Polar Kerr hysteresis loop of the un-annealed CMS (5 nm)/MnGa (30 nm) bilayer.

The typical P-MOKE versus applied magnetic field of the un-annealed CMS (5 nm)/MnGa (30 nm) bilayer is shown in Figure 4 as an example to clarify the magnetization process of the CMS film and MnGa layer. The different shape of the Kerr hysteresis curve from that of the *M*-*H* loop measured by VSM originates from the reflectance phase difference of the light at the bilayer interface in the P-MOKE measurement. The *H*${}_{\mathrm{SW}}$ value is the same as that obtained from the out-of-plane *M*-*H* loop. An inverted P-MOKE hysteresis loop was observed for the un-annealed CMS (5 nm)/MnGa (30 nm) bilayer, which can be attributed to the AFM exchange coupling at the interfaces of the MnGa and CMS films. Such hysteresis has been observed for inhomogeneous films and antiferromagnetically coupled bilayer films [28,29,30]. The magnetization configuration of the MnGa and CMS films at 0 kOe is illustrated in Figure 4. Suppose that the applied magnetic field is sufficiently large in the positive direction; then the magnetization of the MnGa and CMS films are parallel. As the applied magnetic field decreases, the magnetization of CMS starts to rotate from a parallel direction to an antiparallel direction when the applied magnetic field is less than *H*${}_{S-}$. By further reducing the applied magnetic field, the magnetization of MnGa rotates from the up direction to the down direction if the absolute value of the applied magnetic field is sufficiently large to overcome the AFM exchange coupling between the MnGa and CMS films. The P-MOKE hysteresis loop exhibited good PMA properties, which originates from the strong AFM exchange coupling between the MnGa and CMS films.

**Figure 5:**
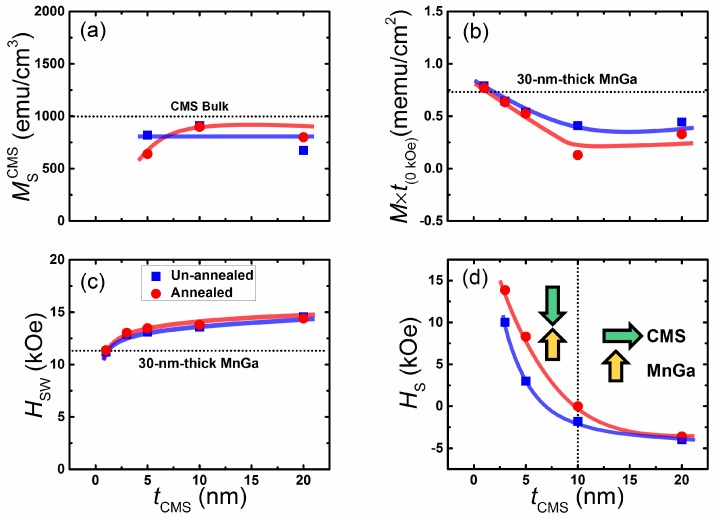
(**a**) Thickness dependence of saturation magnetization for the CMS film ($M_{S}^{\mathrm{CMS}}$); (**b**) Thickness dependence of remnant magnetization ($M\times t_{\left(\mathrm{0kOe}\right)}$); (**c**) switching ($H_{\mathrm{SW}}$); and (**d**) saturation fields in antiparallel state ($H_{S-}$) for CMS/MnGa bilayer films.

The $t_{\mathrm{CMS}}$ dependence of the saturation magnetization of the CMS layer, which is estimated from the out-of-plane *M*-*H* loop of CMS (5–20 nm)/MnGa (30 nm) bilayers, is shown in Figure 5a. The saturation magnetization value of CMS for un-annealed samples with different thicknesses is roughly constant (about 800 emu/cm${}^{3}$), which is comparable with previous studies [31,32]. The saturation magnetization is approximately 600 emu/cm${}^{3}$ for the annealed sample with a 5-nm-thick CMS. By increasing the CMS thickness to 10 nm, the saturation magnetization also increases. In Figure 5b,c, the $t_{\mathrm{CMS}}$ dependence of $M\times t_{\left(\mathrm{0kOe}\right)}$ and $H_{\mathrm{SW}}$ for un-annealed and annealed samples are shown, respectively. For comparison, the $M\times t_{\left(\mathrm{0kOe}\right)}$ and $H_{\mathrm{SW}}$ of 30-nm-thick MnGa is denoted by a dotted line. Notice from these figures that the $t_{\mathrm{CMS}}$ dependence of the $M\times t_{\left(\mathrm{0kOe}\right)}$ value exhibits a similar behavior for un-annealed and annealed samples. The $M\times t_{\left(\mathrm{0kOe}\right)}$ value decreases linearly as the CMS thickness increases up to 10 nm, and then its value saturates for a 20-nm-thick CMS film. Furthermore, a smaller $M\times t_{\left(\mathrm{0kOe}\right)}$ value was observed for samples annealed at 400 ${}^{\circ }$C. The $t_{\mathrm{CMS}}$ dependence of $H_{\mathrm{SW}}$ continues to increase as the CMS film thickness increases for both series samples. The smaller $M\times t_{\left(\mathrm{0kOe}\right)}$ and larger $H_{\mathrm{SW}}$ values of the CMS (1–20 nm)/MnGa (30 nm) bilayer compared with those of 30-nm-thick MnGa suggests an AFM exchange coupling between the MnGa and CMS films.

The $t_{\mathrm{CMS}}$ dependence of $H_{S-}$ for un-annealed and annealed samples is shown in Figure 5d. A positive value of $H_{S-}$ was observed for both un-annealed and annealed samples with a 5-nm-thick CMS film. Indeed, the magnetization of CMS is coupled completely antiferromagnetically with the magnetization of the MnGa film. When the thickness of the CMS film is sufficiently small (on the order of a few nanometers), the exchange coupling interaction is able to align magnetic moments of the CMS film completely antiferromagnetically to magnetic moments of the MnGa film in a perpendicular direction. As the thickness of the CMS film increases and surpasses the critical thickness, the magnetization of CMS tilts from the perpendicular direction to the in-plane. On the other hand, a number of magnetic moments of the CMS film increases as the film thickness increases. We considered the configuration of magnetic moments at interfaces and the upper part of the CMS film. Magnetic moments of the CMS film are aligned in a perpendicular direction at the interface of MnGa and CMS films, whereas they are aligned in an in-plane direction for the upper part of the CMS film. The critical thickness of the CMS film to observe PMA with AFM coupling in CMS/MnGa bilayers is less than 10 nm, which is relatively large compared with previous studies on Co/MnGa [19], Fe/MnGa [20], and Fe-Co/MnGa [21] bilayers. This can be attributed to a strong AFM coupling and moderate saturation magnetization of the CMS film in the CMS/MnGa bilayer.

## 4. Conclusions

To fabricate a synthetic ferrimagnet with good PMA, the thickness of CMS cubic Heusler alloys was varied for un-annealed and annealed CMS (1–20 nm)/MnGa (30 nm) bilayers. XRD results were discussed for the (002) peak of the cubic $L2_{1}$ structure of CMS on the MnGa layer, even for a 3-nm-thick CMS, for both un-annealed and annealed samples. The quality of CMS improved as the CMS thickness increased. The smaller $M\times t_{\left(\mathrm{0kOe}\right)}$ and larger $H_{\mathrm{SW}}$ values of the CMS (1–20 nm)/MnGa (30 nm) bilayer compared with 30-nm-thick MnGa indicated an AFM exchange coupling between MnGa and CMS films for both un-annealed and annealed samples. Annealed samples exhibited smaller $M\times t_{\left(\mathrm{0kOe}\right)}$ values compared with un-annealed samples, which suggests a stronger AFM exchange coupling. The critical thickness for the remaining PMA properties of the bilayer were estimated from magnetic properties. A relatively large critical thickness of less than 10 nm was observed for CMS/MnGa bilayers, which originates from strong AFM coupling and moderate saturation magnetization.
